# Are soluble E-selectin, ICAM-1, and VCAM-1 potential predictors for the development of diabetic retinopathy in young adults, 15–34 years of age? A Swedish prospective cohort study

**DOI:** 10.1371/journal.pone.0304173

**Published:** 2024-06-06

**Authors:** Charlotte Ekelund, Jonatan Dereke, Charlotta Nilsson, Mona Landin-Olsson

**Affiliations:** 1 Diabetes Research Laboratory, Department of Clinical Sciences, Faculty of Medicine, Lund University, Lund, Sweden; 2 Department of Pediatrics, Helsingborg Hospital, Helsingborg, Sweden; 3 Department of Endocrinology, Skåne University Hospital, Lund, Sweden; Medical School, University of Zagreb, CROATIA

## Abstract

The aim of this study was to determine plasma levels of three adhesion molecules that may contribute to the development of diabetic retinopathy; soluble endothelial selectin (sE-selectin), soluble intercellular adhesion molecule-1 (sICAM-1), and soluble vascular cell adhesion molecule-1 (sVCAM-1), in young adults, aged 15–34 years at diagnosis of diabetes, to find potential predictors for development of retinopathy, and to evaluate their relation to diabetes associated autoantibodies. Participants with type 1 (n = 169) and type 2 diabetes (n = 83) were selected from the complications trial of the Diabetes Incidence Study in Sweden and classified in two subgroups according to presence (n = 80) or absence (n = 172) of retinopathy as determined by retinal photography at follow-up 8–10 years after diagnosis of diabetes. Blood samples were collected at diagnosis in 1987–88. The levels of sE-selectin, sICAM-1, and sVCAM-1 were analysed by enzyme-linked immunosorbent assay and islet cell antibodies by a prolonged two-colour immunofluorescent assay. Mean HbA1c (p<0.001) and clinical characteristics: mean body mass index (p = 0.019), systolic blood pressure (p = 0.002), diastolic blood pressure (p = 0.003), male gender (p = 0.026), and young age at diagnosis of diabetes (p = 0.015) remained associated with development of retinopathy in type 1 diabetes. However, in a multivariate analysis only HbA1c remained as a risk factor. sE-selectin was significantly higher in the group with type 2 diabetes and retinopathy, compared to the group with type 2 diabetes without retinopathy (p = 0.04). Regarding sE-selectin, sICAM-1, and sVCAM-1 in participants with type 1 diabetes, no differences were observed between the groups with or without retinopathy. This trial confirmed the role of HbA1c and clinical characteristics as predictors for development of retinopathy in type 1 diabetes. sE-selectin stands out as a potential predictor for development of retinopathy in type 2 diabetes, whereas a predictive role for sICAM-1 and sVCAM-1 could not be identified neither for type 1 nor type 2 diabetes.

## Introduction

The incidence of childhood type 1 diabetes mellitus (T1DM) has increased worldwide with Sweden having the highest incidence in the world next to Finland [1, 2]. Even the burden of type 2 diabetes mellitus (T2DM) in children and adolescents is substantial [3]. Due to severe complications later in life, diabetes mellitus (DM) causes major morbidity and mortality. About a third of people with DM have signs of diabetic retinopathy (DR), and even more for people with T1DM, which can lead to impairment or loss of vision [4, 5]. The prevalence of DR among adolescents and young adults with T1DM and T2DM has been reported as 5.6% and 9.1%, respectively, in the SEARCH study with a mean DM duration of 7.9 years [6], and as 3.4% and 6%, respectively, in a more recent study with a mean DM duration of 4.1 years for T1DM and 1.1 years for T2DM [7], i.e., a declining trend for T1DM due to intensive treatment with optimized glycemic control [8]. There is a concern for increasing prevalence of T2DM and DR. For adolescents and young adults with T2DM with mean DM duration 11 years, the TODAY follow-up study has reported a prevalence of DR as per 51.0% in 2017–2018 compared to 13.7% seven years earlier [9]. Hyperglycemia, DM duration, hypertension, dyslipidemia, puberty, pregnancy, and ethnic origin are important risk factors for development and progression of DR [4, 5, 9–13], but findings regarding gender and body mass index (BMI) have been inconsistent. According to the Diabetes Incidence Study in Sweden (DISS), the prevalence of DR seemed to be higher in men [14], whereas other studies could not show such a difference between the genders [4, 15–18]. Furthermore, DISS has shown an association between BMI and DR among young patients with DM [14] while studies performed in Finland and the US have not [15, 17]. The TODAY study among youth with T2DM showed a lower prevalence of DR in the most severely obese participants, after adjustment for age, hemoglobin A1c (HbA1c) and DM duration [18].

Studies of circulating biomarkers of inflammation and endothelial dysfunction have indicated predictive value for development of DR [19, 20]. Hyperglycemia enhances leucocyte adhesion to the endothelium via upregulation of adhesion molecules such as soluble endothelial selectin (sE-selectin), soluble intercellular adhesion molecule-1 (sICAM-1) and soluble vascular cell adhesion molecule-1 (sVCAM-1) [21, 22]. This inflammatory activity and endothelial dysfunction have been considered of importance in the pathogenesis of DR [23, 24]. Soluble forms of adhesion molecules released by the affected endothelium are detectable in plasma. Increased expression of sE-selectin, sICAM-1, and sVCAM-1 have been found in patients with T1DM/T2DM and DR in comparison with patients without DR [19, 25–27]. In addition, the expression of sE-selectin and sVCAM-1 in patients with T1DM and T2DM seemed to increase with progression of DR and could thus relate to the severity of DR [28, 29]. A meta-analysis has revealed increased levels of sICAM-1 in patients with DR irrespective of type of diabetes as well as a possible association with the severity of DR [30]. In a prospective study of inflammatory biomarkers at diagnosis of DM and risk of DR in T1DM in the Diabetes Control and Complications Trial (DCCT), sICAM-1 may have been associated with the development of retinal hard exudates, but neither sICAM-1 nor sVCAM-1 were significantly associated with progression of DR [31]. Another prospective study, investigating the DCCT/Epidemiology of Diabetes Interventions and Complications study (EDIC) cohort and risk of DR in T1DM, has reported an association between increased levels of sE-selectin at diagnosis of DM and development of DR, but not for sICAM-1 and sVCAM-1 [32]. Thus, previous studies have been contradictory. In a study of T2DM patients, no differences could be found in the levels of sE-selectin, sICAM-1, and sVCAM-1 between patients with and without DR, and no associations to progression of DR were identified [33]. In a group with T2DM and DR, sE-selectin was not significantly higher neither compared to patients with T2DM without DR, nor to healthy control subjects [34]. As previously mentioned, sE-selectin has been associated with progression of DR in patients with T2DM [29]. However, yet another study in T2DM showed that this association was not independent of glycemic control and BMI [35].

Published data reflect the presence of DR and high levels of soluble adhesion molecules, although with inconsistencies. Due to the role of adhesion molecules in endothelial activation, their increased levels could potentially indicate the risk for, and severity of, DR development. The primary aim of this study was to determine plasma levels of sE-selectin, sICAM-1, and sVCAM-1 in young adults at diagnosis of DM, to find potential predictors for the development of DR. The secondary aim was to evaluate the adhesion molecules’ relation to diabetes associated autoantibodies in the complications trial of the Diabetes Incidence Study in Sweden (K-DISS) cohort. If an association between sE-selectin, sICAM-1, and/or sVCAM-1 and DR can be identified, and hence these biomarkers could be used at an early stage for prediction of DR, high-risk patients for the development of DR could be recognised. Healthcare could then be individualised with resources and screening programmes focusing on patients with the biggest needs. Hopefully, the onset of DR can be prevented or delayed, providing benefits not only for the individual but also for society and the payers.

## Materials and methods

### The Diabetes Incidence Study in Sweden (DISS)

Since 1983, a nationwide population-based prospective study, DISS, registers the incidence of DM, except gestational diabetes, and its complications in the age group 15–34 years. The objective of DISS is to identify factors relevant for the development of DM and its complications [36]. Data from DISS have been used in the sub study K-DISS, previously described in detail, to investigate the prevalence of DR [14] and diabetic kidney disease [37, 38] at follow-up. Part of those results were used in this study [14].

### Study population

**Fig 1:**
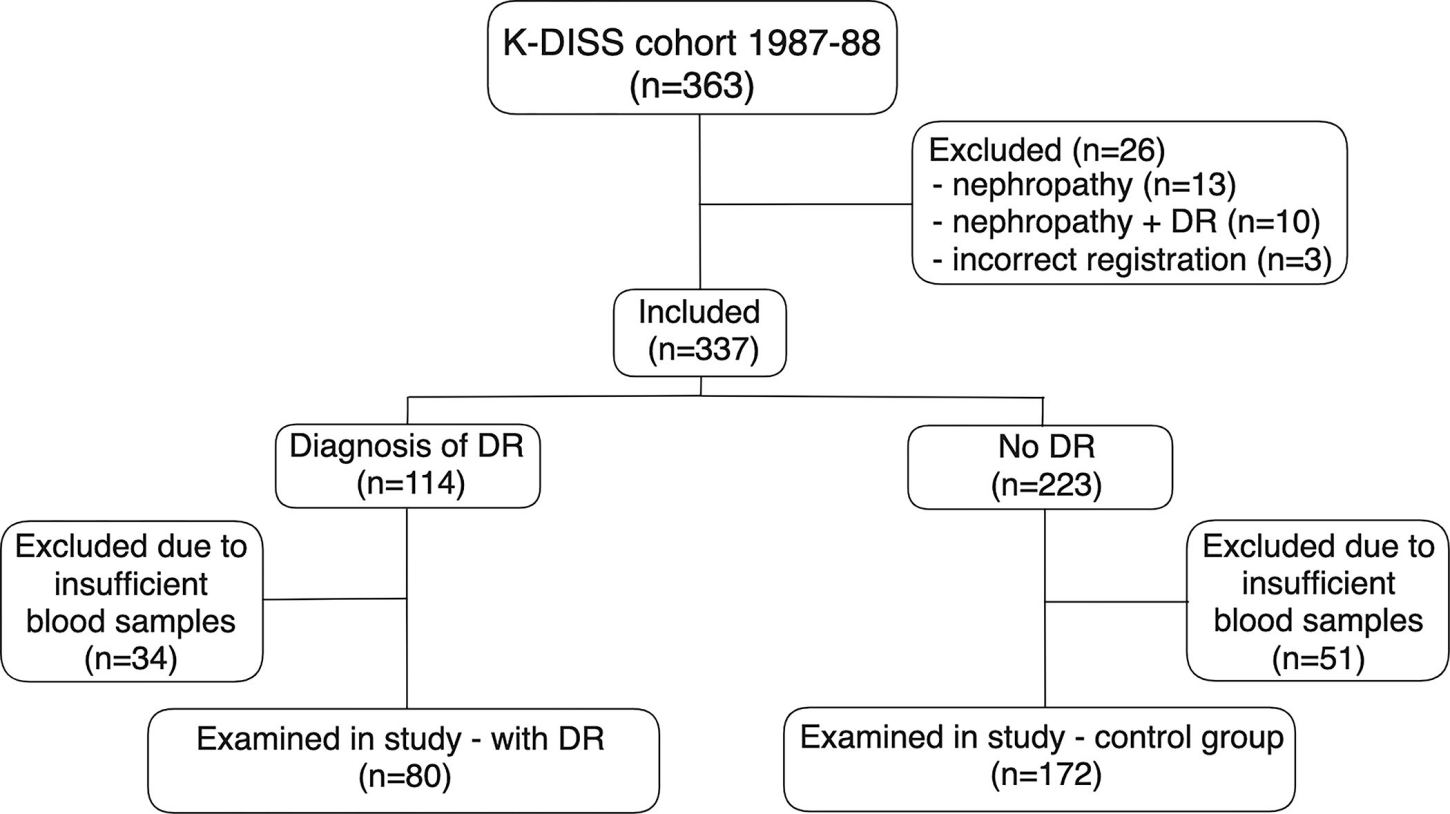
The recruitment process of study participants. K-DISS, complications trial of the Diabetes Incidence Study in Sweden; DR, diabetic retinopathy.

The study population consisted of all patients (n = 363), participating in K-DISS, diagnosed by clinicians as T1DM (n = 226) or T2DM (n = 137), according to the World Health Organization criteria [39], in 1987–88 at an age of 15–34 years. The classification of T1DM was made by islet cell antibodies (ICA) and/or clinical characteristics and patients presenting without ICA were classified as T2DM. Patients who developed nephropathy (n = 13) or concomitant nephropathy and DR (n = 10) during follow-up were subsequently excluded in order not to risk investigating patients with nephropathy caused by conditions unrelated to DM. Three patients were excluded due to incorrect registration in the registry. At follow-up 8–10 years after diagnosis of DM, 114 out of 337 participants had developed any grade of DR, but no other complications. The remaining 223 participants without DR, or any other complications, constituted the control group. 34 blood samples in the group with DR and 51 blood samples in the control group contained insufficient volume of blood for the tests to be conducted. Hence, in total 80 participants in the group with DR and 172 participants in the control group were investigated. Classification of DR was performed by dilated retinal photography in most cases, by ophthalmoscopy or slit-lamp bio-microscopy data from medical records if no photographs had been taken, and the 11-step scale of the alternative classification of the Wisconsin study [14, 40]. The median number of eye examinations by photography was three per participant, without any difference between T1DM and T2DM. All photographs taken since diagnosis of DM were centrally assessed by two independent and experienced graders. The severity of DR was determined by the eye most severely affected. Height and weight for calculation of BMI, as well as systolic and diastolic blood pressure (BP), were reported at follow-up. All HbA1c values since diagnosis were requested. Tobacco use was defined as current or previous daily use. The recruitment process of study participants is described in Fig 1.

### Analyses

#### Sample handling

At diagnosis of DM blood samples were collected in tubes containing ethylenediaminetetraacetic acid (EDTA) and sent by mail from hospitals and primary health care centres in Sweden to the central laboratory for analyses. Plasma was separated by centrifugation at 2000 x *g* and stored at a temperature of -80°C until analysis of the adhesion molecules. ICA were determined, in serum samples collected at diagnosis of DM, by a prolonged two-colour immunofluorescent assay at Malmö General Hospital [41, 42], and have previously been reported [43]. HbA1c was measured at local laboratories using ion-exchange chromatography and presented as a percentage as per the National Glycohemoglobin Standardization Program (NGSP) and later converted to mmol/mol as per the International Federation of Clinical Chemistry (IFCC) units. The mean HbA1c value during the entire follow-up period was calculated for each participant.

#### Laboratory analyses

The plasma concentrations of the soluble adhesion molecules were determined using the enzyme-linked immunosorbent assay ELISA DuoSet (R&D Systems, Minneapolis, MN, USA), according to the manufacturer’s instructions. Plasma samples were diluted 1:25 for sE-selectin, 1:200 for sICAM-1, and 1:1000 for sVCAM-1. Samples from participants with and without DR were alternated on each ELISA microplate to reduce interassay variability and measured in duplicate. Two in-house plasma controls were added to each run. For absorbance measurements at 450 nm and 580 nm, a FLUOstar Optima Microplate Reader (BMG LABTECH, Ortenberg, Germany) was used. A four-parametric logistic regression standard curve was used to calculate the concentrations. In this study, the intraassay coefficients of variation were 2.8% for sE-selectin, 4.3% for sICAM-1, and 3.3% for sVCAM-1. The lower detection limits of the assays were 1.5 ng/ml for sE-selectin, 5.1 ng/ml for sICAM-1, and 33.8 ng/ml for sVCAM-1.

### Ethics

The study was approved by the Regional Ethical Review Board, Lund, Sweden, (LU 176–03 and LU 2009–423) and performed in accordance with the Declaration of Helsinki. Written information about the objective of the study was provided before inclusion and each participant gave written informed consent. For minors, written informed consent was obtained from parents or guardians. The authors had access to information that could identify individual participants after data collection. Data were accessed 26-Sep-2016 for research purposes.

### Statistical analyses

All HbA1c values since diagnosis of DM were used when calculating the mean HbA1c using the area under the curve of HbA1c over time to compensate for the occasionally irregular intervals between the measurements.

The D’Agostino-Pearson test was used to examine normal distribution of data. Results that were normally distributed are presented as mean ± standard deviation (SD). The t-test was used for comparison between groups. The non-parametric variables, i.e., results not normally distributed, are presented as median and interquartile range and the Mann-Whitney U test was used for comparison between groups. Frequencies are presented as numbers and percent and were compared using the chi-squared test. Correlations between variables were determined using Spearman’s rank correlation test and presented with 95% confidence intervals (CI). Logistic regression was used to analyse the effect of several independent variables on DR. Only statistically significant variables were included in a multivariate analysis model. Statistical analyses were performed using MedCalc for Windows (version 12.7.0.0, MedCalc Software, Ostend, Belgium). P-values below 0.05 were considered statistically significant.

## Results

In the study population of 252 participants, 80 (31.7%) had developed DR and 172 (68.3%) had not developed DR 8–10 years after diagnosis of DM. Participants with DR had statistically significant higher mean HbA1c (70.4±15.2 vs. 60.7±13.8 mmol/mol; p<0.0001), mean BMI (24.7(23.1–27.0) vs. 24.0(21.6–26.5) kg/m^2^; p = 0.03), systolic BP (124±10.5 vs. 120±11.9 mm Hg; p = 0.02) and diastolic BP (76.9±8.21 vs. 74.0±8.03 mm Hg; p = 0.009) compared to participants without DR. There were also significant differences in age at diagnosis of DM and gender where participants with development of DR were younger at onset (23.5±5.39 vs. 25.2±5.52 years; p = 0.03) and the proportion of men was higher (65.0 vs. 50.6%; p = 0.03). No significant differences were found between the groups (DR vs. no DR) regarding the type of DM, (T1DM 65.0 vs. 68.0%; p = 0.64), the levels of ICA (48.0(0–1000) vs. 128(0–1000) Juvenile Diabetes Foundation (JDF) units; p = 0.40) or tobacco use (50.0 vs. 45.8%; p = 0.54). No significant differences were found between the groups (DR vs. no DR) regarding the levels of the adhesion molecules sE-selectin (10.4(7.64–12.5) vs. 9.33(6.80–12.2) ng/ml; p = 0.25), sICAM-1 (109(80.3–153) vs. 106(73.3–150) ng/ml; p = 0.93) or sVCAM-1 (490±154 vs. 455±157 ng/ml; p = 0.09).

Significant negative correlations were found between sICAM-1 and ICA (r_s_ = -0.19; p = 0.002), and sVCAM-1 and ICA (r_s_ = -0.13; p = 0.04). No correlation was found between sE-selectin and ICA. No correlation was found between the levels of ICA and age at diagnosis of DM.

In a logistic regression model with DR as the dependent variable and mean HbA1c, mean BMI, systolic BP, diastolic BP, gender, and age at DM diagnosis as independent variables, all lost statistical significance except mean HbA1c (Odds Ratio 1.04, 95% CI 1.02–1.07; p = 0.0001).

Participants with T1DM who had developed DR 8–10 years after diagnosis of DM had statistically significantly higher mean HbA1c (p<0.001), mean BMI (p = 0.019), systolic BP (p = 0.002) and diastolic BP (p = 0.003), but lower age at diagnosis of DM (p = 0.015) compared to participants without DR. The proportion of men was higher among those with T1DM and DR, as compared to no DR (p = 0.026). Regarding sE-selectin, sICAM-1, and sVCAM-1 in participants with T1DM no differences were observed between the groups with or without DR.

Among participants with T2DM, no statistically significant differences regarding mean HbA1c and clinical data were found between those with development of DR and without. Mean HbA1c was higher in the group with DR but did not reach statistical significance (p = 0.08). However, sE-selectin, but not sICAM-1 or sVCAM-1, was significantly higher in the group with T2DM and DR, as compared to no DR (11.8(9.97–12.6) vs. 9.43(5.85–12.7) ng/ml; p = 0.04).

Clinical and biochemical data for study participants with T1DM and T2DM with and without development of DR are presented in Table 1.

**Table 1:** Clinical and biochemical data for study participants with type 1 and type 2 diabetes mellitus, aged 15–34 years at diagnosis of diabetes, with and without development of diabetic retinopathy 8–10 years after diagnosis of diabetes.

	T1DM (n = 169)			T2DM (n = 83)		
	Retinopathy (n = 52)	No retinopathy (n = 117)	P-value	Retinopathy (n = 28)	No retinopathy (n = 55)	P-value
**At diagnosis of DM**						
Age (years)	22.9±5.27	25.1±5.53	p = 0.015	24.8±5.48	25.4±5.53	p = 0.65
Gender (male/female)	35/17 (67.3%)	57/60 (48.7%)	p = 0.026	17/11 (60.7%)	30/25 (54.5%)	p = 0.59
ICA (JDF units)	512 (96–2000)	1000 (112–2000)	p = 0.43			
sE-selectin (ng/ml)	9.19 (6.53–12.5)	9.18 (7.11–12.1)	p = 0.83	11.8 (9.97–12.6)	9.43 (5.85–12.7)	p = 0.04
sICAM-1 (ng/ml)	103 (73.4–147)	102 (66.7–135)	p = 0.90	123 (94.8–178)	127 (84.4–185)	p = 0.96
sVCAM-1 (ng/ml)	456 (370–578)	446 (355–528)	p = 0.32	489 (420–617)	479 (371–574)	p = 0.24
**At follow-up**						
Mean BMI (kg/m^2^)	24.1 (22.7–26.8)	23.3 (21.3–25.6)	p = 0.019	25.8 (23.7–27.2)	24.9 (22.9–28.6)	p = 0.84
Systolic BP (mmHg)	126±9.55	120±11.8	p = 0.002	120±11.2	120±12.4	p = 0.89
Diastolic BP (mmHg)	77.7±8.18	73.6±7.92	p = 0.003	75.4±8.19	74.7±8.30	p = 0.72
Tobacco use (yes/no)	23/27 (46%)	50/61 (45%)	p = 0.91	16/12 (57.1%)	26/29 (47.3%)	p = 0.40
Mean HbA1c IFCC (mmol/mol)	71.6±14.2	60.4±12.5	p<0.001	68.3±17.0	61.2±16.2	p = 0.08

The data are presented as mean ± SD, median (interquartile range) or number (percent).

DM, diabetes mellitus; T1DM, type 1 diabetes; T2DM, type 2 diabetes; ICA, islet cell antibodies; JDF, Juvenile Diabetes Foundation; BMI, body mass index; BP, blood pressure; IFCC, International Federation of Clinical Chemistry.

Participants with T1DM had significantly lower mean BMI than participants with T2DM (p<0.001), but altogether no other differences in clinical data were found when comparing the two groups. Nevertheless, sICAM-1 was statistically higher in the group with T2DM (p = 0.001), whereas no statistically significant differences were found between T1DM and T2DM regarding sVCAM-1 (p = 0.07) and sE-selectin (p = 0.32). Clinical and biochemical data for study participants with T1DM and T2DM are presented in Table 2.

**Table 2:** Comparison of clinical and biochemical data for study participants with type 1 and type 2 diabetes mellitus at diagnosis of diabetes and at follow-up 8–10 years after diagnosis of diabetes.

**At diagnosis of DM**	**T1DM (n = 169)**	**T2DM (n = 83)**	**P-value**
Age (years)	24.4±5.54	25.2±5.48	p = 0.31
Gender (male/female)	92/77 (54.4%)	47/36 (56.6%)	p = 0.74
sE-selectin (ng/ml)	9.18 (7.04–12.2)	10.2 (7.00–12.6)	p = 0.32
sICAM-1 (ng/ml)	103 (67.2–138)	127 (88.5–181)	p = 0.001
sVCAM-1 (ng/ml)	450 (356–544)	479 (405–587)	p = 0.07
**At follow-up**			
Mean BMI (kg/m^2^)	23.7 (22.0–26.0)	25.1 (23.2–27.7)	p<0.001
Systolic BP (mmHg)	122±11.4	121±11.9	p = 0.31
Diastolic BP (mmHg)	74.9±8.20	74.9±8.22	p = 0.98
Tobacco use (yes/no)	73/88 (45.3%)	42/41 (50.6%)	p = 0.44
Mean HbA1c IFCC (mmol/mol)	64.1±14.0	63.7±16.7	p = 0.87

The data are presented as mean ± SD, median (interquartile range) or number (percent).

DM, diabetes mellitus; T1DM, type 1 diabetes; T2DM, type 2 diabetes; BMI, body mass index; BP, blood pressure; IFCC, International Federation of Clinical Chemistry.

## Discussion

Since 1983, a nationwide population-based prospective study, registers the incidence of DM and its complications in the age group 15–34 years in Sweden. In the present study, we investigated the K-DISS baseline values of soluble adhesion molecules and subsequent development of DR 8–10 years after diagnosis of DM, in a cohort diagnosed with DM as young adults in 1987–88. The main finding of this study was that plasma levels of sE-selectin at diagnosis of DM were significantly higher in participants with T2DM who had developed DR 8–10 years after diagnosis of DM compared to participants with T2DM who had not. sE-selectin can therefore be a candidate for identification of T2DM patients at high risk of developing DR. Our research indicates that the levels of sE-selectin may serve as a predictive biomarker for the development of DR in patients with T2DM, similar to the results in the DCCT/EDIC regarding patients with T1DM [32]. Increased levels of sE-selectin have previously been shown to be associated with insulin resistance/hyperinsulinemia [44] which may support our results with significantly increased levels of sE-selectin, but not sICAM-1 and sVCAM-1, in the group with DR in T2DM.

Matsumoto et al. has determined sE-selectin, sICAM-1, and sVCAM-1 in participants with T2DM grouped by presence or absence of DR and reported significantly higher levels of all three adhesion molecules independent of HbA1c in participants with DR [19]. In our study, a predictive role for sICAM-1 and sVCAM-1 at diagnosis of DM regarding development of DR could not be identified neither for T1DM nor T2DM, which is in accordance with the findings in the DCCT/EDIC, where the expressions of sICAM-1 and sVCAM-1 could not predict initiation or progression of DR in T1DM [32]. On the other hand, a large study by the EURODIAB Prospective Complications Study group has shown positive associations with DR for both sE-selectin and sVCAM-1 in T1DM [25]. In the same study, a high correlation was found between sE-selectin and HbA1c indicating that sE-selectin expression on the endothelium was influenced by glycemic control. The inflammatory activity associated with T1DM has been shown to be partly induced by hyperglycemia through adhesion molecules and endothelial dysfunction [45]. To reduce the risk of developing DR in DM, strategies must focus on the improvement of endothelial function as well as control of conventional risk factors.

HbA1c is a well-known predictive biomarker for development of DR [10, 11, 13]. Prior research has demonstrated the impact of early glycemic control on prevention of DR and diabetic kidney disease in childhood-onset T1DM [46]. This study confirmed that suboptimal glycemic control over the course of the disease, measured as mean HbA1c, is a strong predictor for development of DR in T1DM. The prevalence of DR in the cohort was high, 31.7%, even though the participants were intensively treated and used self-glucose monitoring during the follow-up period. Patients with DM may develop microvascular complications despite reasonable glycemic control, indicating the importance of other risk factors. Altogether, we found that the clinical characteristics suboptimal glycemia during follow-up, higher mean BMI, higher systolic and diastolic BP, male gender, and younger age at DM diagnosis were associated with development of DR in T1DM, but not T2DM. However, in a multivariate analysis only mean HbA1c remained as a risk factor. A correct classification of DM is important in this age group. As previously shown, young patients with T2DM have a higher prevalence of severe DR, i.e., moderate or severe non-proliferative DR and proliferative DR, than young patients with T1DM [14]. Regularly performed dilated eye examinations in DM are needed according to treatment guidelines [47, 48].

In this study, higher mean BMI was significantly associated with the development of DR in T1DM and participants with T1DM had significantly lower BMI than participants with T2DM. The association between higher BMI and an increased risk of developing DR has previously been reported [12].

There is no clear gender difference in the development of DR. In this cohort of adolescents and young adults, male gender was associated with development of DR in T1DM. In the Wisconsin Epidemiologic Study of Diabetic Retinopathy XXII, being male was significantly associated with progression of DR in T1DM, independent of other risk factors [12]. However, a higher prevalence of DR in T1DM has been reported for adolescent girls compared to boys in an Australian study [49]. Among American youth with T2DM, males were at higher risk of developing DR compared to females [50], which could not be confirmed in our study. The difference between genders and the development of DR in prior research might depend on the age of onset of DM or hormonal differences during puberty. In the cohort, aged 15–34 years at diagnosis, younger age at diagnosis of T1DM was associated with development of DR. This is consistent with a study reporting higher risk of vascular complications in those living with DM during puberty, compared to young people developing DM after puberty [51]. Therefore, it is important to screen for early signs of DR and thereby identifying risk factors that can be addressed during adolescence.

Incident participants with T1DM in DISS have shown a significant decrease in the frequency of ICA, but not in the levels of ICA, with increasing age, particularly in men [43]. In accordance, this sub study did not show any correlation between the levels of ICA and age at diagnosis of T1DM. However, significant negative correlations were found between sICAM-1 and ICA, and between sVCAM-1 and ICA. On the contrary, other studies have shown a positive correlation [52] or no correlation [53] between sICAM-1 and ICA in patients with T1DM. Whether the adhesion molecules are independent of ICA status or not is still elusive and needs further investigations.

This prospective study has several strengths. It is a nationwide and population-based study with a well-defined cohort of young adults with DM and a long follow-up period of 8–10 years, making it possible to longitudinally identify development of microvascular complications. ICA were used to accurately classify between T1DM and T2DM. Furthermore, patients who developed nephropathy or concomitant nephropathy and DR during follow-up were excluded in order not to risk investigating patients with nephropathy caused by conditions unrelated to DM. Commercially available assays with high sensitivity and low variation were used.

The limitations of our study should also be mentioned. Only baseline levels of the adhesion molecules were measured for the study participants, and the results were consequently restricted to a single measurement of those. It is possible that some degradation may have occurred due to long storage time although the adhesion molecules measured have been shown to remain stable in stored specimens. The exclusion of study participants due to insufficient blood samples in the group with DR and the group without DR were 30% and 23%, respectively. The samples had been used in previous studies, and some of the tubes did no longer contain large enough blood volume for the tests to be conducted. The study focused on adolescents and young adults diagnosed with T1DM and T2DM. However, the majority of those who develop T2DM are diagnosed later in life. Therefore, a broader age range could impact the results. The length of the time interval between onset and diagnosis of T1DM and T2DM may differ and hence, the diabetes duration, with T2DM often having a longer asymptomatic period before diagnosis and therefore a higher risk of microvascular complications at diagnosis and time of inclusion in the study. Although this study has a small number of participants with onset of T2DM in adolescence and early adulthood (n = 83), the results are interesting, since the number of patients with T2DM is increasing globally with diagnosis being made at younger and younger ages.

The results of different published investigations are varying, indicating the potential presence of unidentified factors that may influence the levels of adhesion molecules observed in DR. Chronic inflammation is believed to play a role in the pathogenesis of DR, yet the precise mechanism remains unclear. Our findings do not explain the variation in results reported in prior research, but illustrate a possible association between adhesion molecules in endothelial activation and DR. Further research aiming at defining biomarkers able to identify patients at high risk of developing DR already at diagnosis of DM is essential. Non-invasive, accurate, and cost-effective biomarkers would be needed to help reducing the incidence and progression of DR. Until better biomarkers are available, we must adhere to current treatment guidelines. Enhanced screening for T2DM, improved management of DM and improved screening and treatment of DR have all contributed to a decline in both incidence and severity of DR. This underscores the importance of early intervention and efficient treatment, as target tissues appear to retain a memory of poor metabolic control, even many years later, currently known as the “legacy effect” [54].

In conclusion, there was a significant association between increased plasma levels of sE-selectin at diagnosis of T2DM and development of DR within 10 years. The clinical characteristics: suboptimal glycemia, higher BMI, higher systolic and diastolic BP, male gender, and younger age at DM diagnosis for young adults predicted development of DR in T1DM, but not T2DM.

The results from this study encourage further exploration in larger populations and with longer follow-up periods to uncover new biomarkers for early prediction of DR, facilitating tailored and timely interventions from diagnosis of DM for high-risk patients.
